# Rupture of Both Left-Sided Valves Following Endocarditis Causing One Trouble After Another: A Case Report

**DOI:** 10.7759/cureus.21189

**Published:** 2022-01-13

**Authors:** Mtanis Khoury, Marco Khiella, Gaurav K Sharma, Wasey Ali Yadullahi Mir, Daniela Kovacs, Sandeep Khosla, Vijay Ketan Reddy, Steven Monahan, Sharada KC, Dhan B Shrestha

**Affiliations:** 1 Internal Medicine, Mount Sinai Hospital, Chicago, USA; 2 Internal Medicine, Rosalind Franklin University of Medicine and Science, Chicago, USA; 3 Internal Medicine, Mount Sinai Medical Center, Chicago, USA; 4 Cardiology, Rosalind Franklin University of Medicine and Science, Chicago, USA; 5 Medicine, Mount Sinai Hospital, Chicago, USA

**Keywords:** intravenous drug abuse, male, heart failure, infective endocarditis, mitral valve insufficiency

## Abstract

Despite the advances in managing left-sided infective endocarditis, complications are still not uncommon. Both aortic and mitral insufficiency can occur from infective endocarditis. In addition, valvular insufficiency due to rupture of valves presents acutely with cardiac decompensation and requires early surgical intervention. Here, we report a case of a 38-year-old intravenous drug user male with Group A *Streptococcus-*associated left-sided native valve infective endocarditis who presented with acute heart failure three months after his treatment of infective endocarditis. Infective endocarditis complications can lead to severe valve damage, causing acute heart failure, and may require immediate surgical intervention.

## Introduction

Acute aortic insufficiency can occur due to multiple causes, including endocarditis, aortic dissection, traumatic rupture, and after valve replacement. The former two happen to be the most common causes [1]. Acute occurrence of aortic insufficiency causes a quick decline in left ventricular diastolic volume and stroke volume. It does not provide enough time for the compensatory mechanisms of the heart to come into play. As a result, cardiac decompensation occurs early in acute insufficiency, and the patient develops heart failure and pulmonary edema. Thus, it is a medical emergency.

Acute mitral insufficiency occurs due to damage to the components or supporting structures of the mitral valve. It commonly occurs due to ischemic damage to the papillary muscle in acute myocardial infarction. However, it also can occur due to non-ischemic disease processes like infective endocarditis, myxomatous degeneration, trauma, or rheumatic heart disease. Acute mitral regurgitation also presents with acute pulmonary edema and cardiac decompensation as the sudden hemodynamic changes strain the heart [2].

Here, we present a case of rupture of both aortic and mitral valves in a patient who had completed antibiotic therapy for infective endocarditis. The patient underwent the replacement of both valves.

## Case presentation

A 38-year-old male with a history of tobacco use disorder and past intravenous (IV) drug use, currently in a methadone program, presented initially with recurrent non-bilious non-blood-stained vomiting associated with diffuse epigastric pain. He also had bilateral lower limb swelling and paroxysmal nocturnal dyspnea. Furthermore, his appetite had decreased over a week before the presentation. He did not complain of chest pain, palpitations, orthopnea, fever, chills, and changes in bowel or bladder habits.

**Figure 1:**
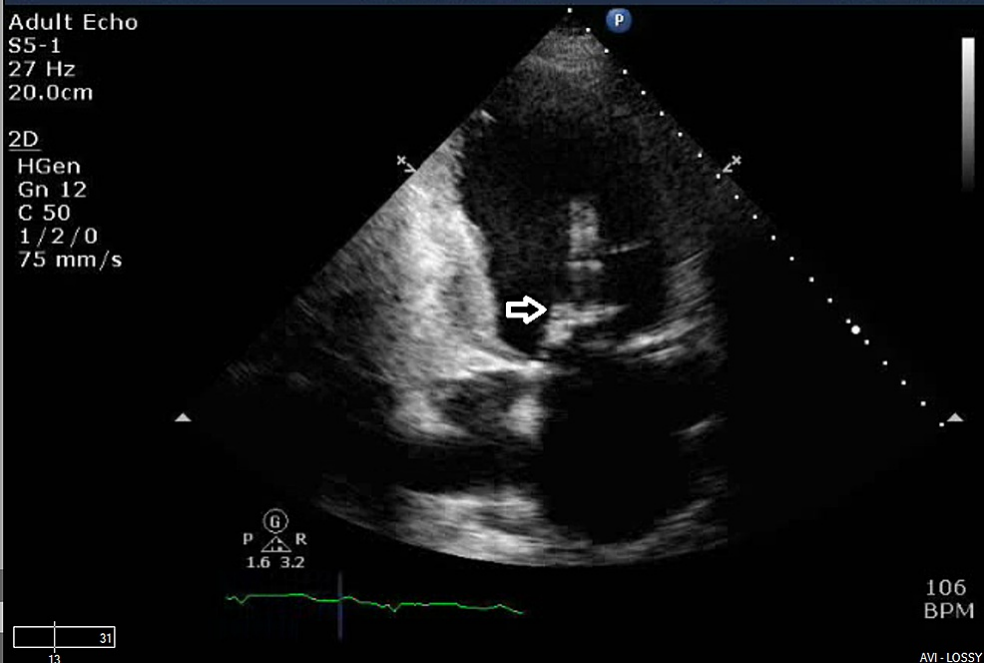
Two-chamber view showing vegetation on the anterior leaflet of mitral valve

Three months before presentation, the patient had presented with features of sepsis and was diagnosed with infective endocarditis with mitral valve involvement and blood culture positive for Group A *Streptococcus* (GAS). Transesophageal echocardiography at that time showed an ejection fraction (EF) of 55%-60% and small fixed vegetation on the anterior leaflet with perforation of the A1 region of the anterior leaflet of the mitral valve with mild regurgitation (Figure 1). Other valves did not show any vegetation. He had multiple abscesses in the sacrum, coccyx, left thigh, and left hip during the episode. He underwent debridement of the wounds. He was subsequently treated with IV ceftriaxone for six weeks for endocarditis and IV vancomycin for one week for infected ulcers.

He was afebrile on presentation, with a heart rate in 100-110s, blood pressure of 113/58 mm Hg, and oxygen saturation in the 90s on room air. Examination revealed bounding pulses, a diastolic murmur, pedal edema, and bibasilar crackles. His leukocyte and platelet counts were normal, with 11 mg/dL hemoglobin. He had a serum creatinine of 2 mg/dL, elevated brain natriuretic peptide, hyponatremia, and normal troponin levels. His liver function tests revealed total bilirubin of 1.8 mg/dL, aspartate aminotransferase (AST) of 728 units per liter, alanine aminotransferase (ALT) of 529 units per liter, a prothrombin time international normalized ratio of 3.3 with normal alkaline phosphatase levels.

**Figure 2:**
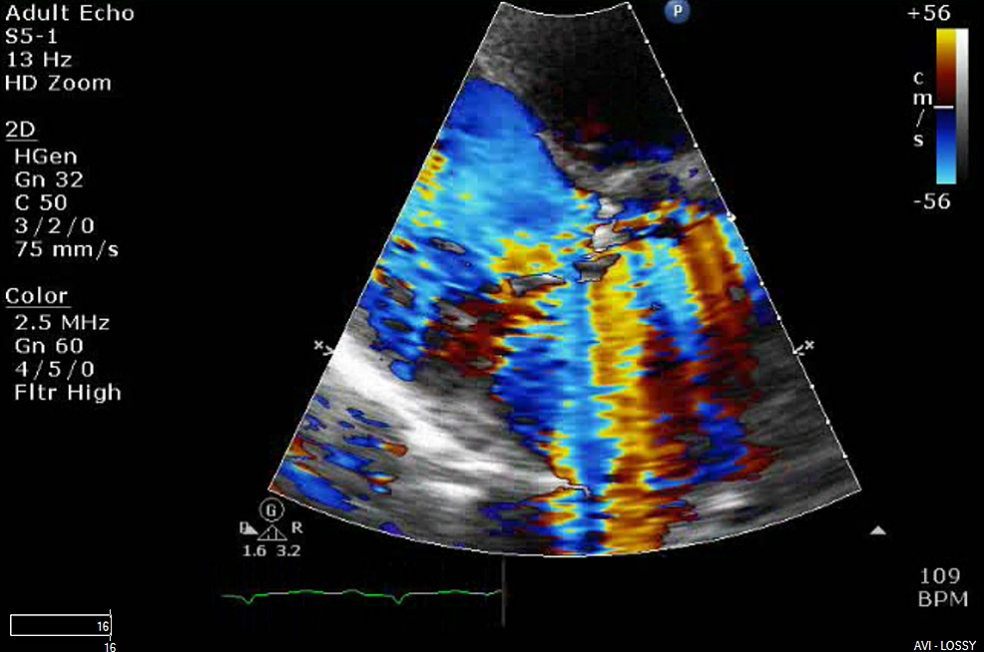
Aortic valve long-axis view showing severe aortic regurgitation with ruptured valve

**Figure 3:**
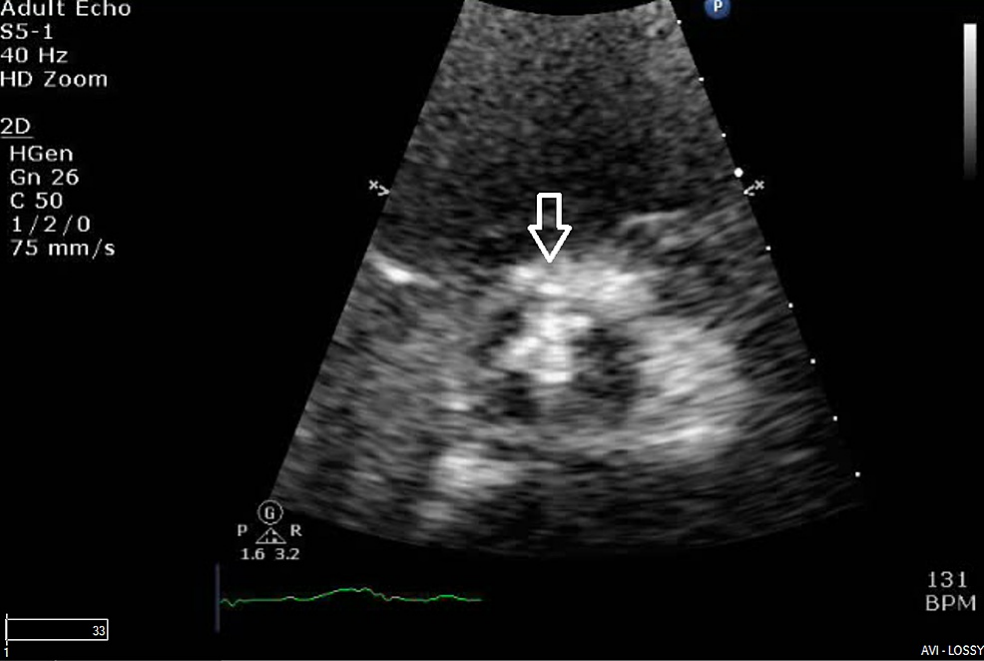
Aortic valve in the short axis view showing vegetation on all three cusps

Subsequently, a chest x-ray was done, which revealed pulmonary edema. An echocardiogram was performed, which showed EF - 35%-40% and dilatation of both atria. Mitral valve had a severe regurgitation with an eccentric, posteriorly directed jet and vegetation on the anterior mitral valve (as seen in prior echocardiogram). The aortic valve had ruptured with wide-open regurgitation with aortic jet covering more than 65% of the left ventricular outflow tract area with vegetations on cusps (Figures 2, 3). Pressure half-time through the aortic valve was 138 m/s, and pulmonary artery systolic pressure had increased to 47 mm Hg. A lower extremity duplex ultrasound showed a right distal deep venous thrombosis, and he was started on therapeutic enoxaparin.

Cardiothoracic surgery was consulted. He then underwent a cardiopulmonary bypass surgery with median sternotomy. Intraoperatively, a destroyed aortic valve with evidence of perforation of the left coronary leaflet and vegetation measuring 1 cm on that leaflet was observed. The mitral valve had small vegetations and was also destroyed. Both the valves were replaced with a 21-mm tissue aortic valve and a 31-mm mitral tissue valve. Two chest tubes and a drain were kept in the mediastinum, and temporary biventricular pacing wires were placed.

The patient’s hemoglobin decreased to 6.1 mg/dL during the hospital stay, and platelets dropped to 34,000 per microliter. He received six units of packed red blood cells and seven units of platelet transfusion.

He was maintained on dobutamine, nor-epinephrine, IV ceftriaxone, and vancomycin postoperatively. The intraoperative samples from deep wounds did not grow any organism on bacterial or fungal cultures.

The histopathology report of the mitral and aortic valve showed acute endocarditis, fibrin deposition, and calcification with the possibility of preexisting rheumatic heart disease. Additionally, his postoperative period was complicated by recurrent pleural effusion and retrosternal hematoma. Therefore, he underwent a video-assisted thoracoscopy with pleural decortication. Subsequently, an echocardiogram was performed. Left ventricular EF was 15%-20%. Mitral valve bioprosthesis was in place with a peak velocity of 1.89 m/s, mean gradient of 5 mm Hg, pressure half-time of 85 msec, consistent with normally functioning prosthesis with normal sewing ring showing no rocking motion and no evidence of dehiscence. Aortic valve bioprosthesis was also in place with a transvalvular velocity within normal range, peak velocity of 2.7 m/s consistent with normally functioning prosthesis with normal sewing ring showing no rocking motion and no evidence of dehiscence.

Currently, till six months follow-up, the patient is fine without any complications and has been symptomatically better with rehabilitation.

## Discussion

Infective endocarditis refers to the infectious process affecting the heart's endocardium, including the intracardiac devices. Infective endocarditis can be acute or subacute based on the onset and duration of illness [3]. The microorganisms responsible include *Staphylococcus aureus*, viridans group of *Streptococci, Enterococci,* coagulase-negative *Staphylococci,* HACEK group (*Haemophilus, Actinobacillus, Cardiobacterium, Eikenella,* and *Kingella*). *S. aureus *and the viridans group of* Streptococci* seem to be the most common agents in community-acquired infectious endocarditis [4,5]. Our patient was diagnosed with GAS infective endocarditis. GAS, also known as *Streptococcus pyogenes*, is an uncommon cause of endocarditis. It causes invasive diseases, including infective endocarditis in people with drug abuse and people with homelessness [6,7]. The history of intravenous drug abuse predisposed our patient to GAS endocarditis.

IV drug abuse causes right-sided infective endocarditis in most cases. However, left-sided endocarditis is also common. In IV drug abusers, left-sided endocarditis often occurs in people with older age and those with other underlying cardiac conditions [8]. Furthermore, *S. aureu*s seems to be the common cause of right-sided endocarditis, whereas *Streptococci *and *Enterococci* are common causes of left-sided endocarditis in people with IV drug use [8].

Infective endocarditis causes various cardiac as well as non-cardiac complications. Our patient sees that left-sided endocarditis can lead to septic embolization, leading to abscesses or other complications [3]. The endocardial involvement and vegetations in infective endocarditis directly affect the valve leaflets leading to insufficiency of the valves. Additionally, perivalvular abscesses, especially in the case of the aortic valve, can lead to aortic insufficiency. The spread of infection to the mitral valve through direct extension or from a regurgitant jet of the infected aortic valve can lead to mitral aneurysm [9,10]. In our case, the effects of endocarditis in the valves with vegetation and perforation were the reason for severe acute insufficiency of both valves.

Acute severe regurgitations which lead to heart failure should be managed by surgical intervention as soon as possible [11]. In patients with active infective endocarditis, valve repair seems to be a better option than replacement [12] as valve replacement is associated with the risk of future episodes of endocarditis [13]. However, our patient was not in active infection and underwent both mitral and aortic valves replacement. Although transcatheter valve replacements are widely used, there is limited data on transcatheter valve replacement for multiple valves, and a surgical approach was used [14].

Valve replacement is associated with multiple complications like postoperative bleeding, systemic embolization, valve migration, insufficiency, renal failure, and cardiogenic shock [15]. At present, at six months follow-up, our patient has a fully functioning prosthetic valve and is following rehabilitation to regain premorbid functionality.

## Conclusions

Valves can undergo substantial damage with episodes of infective endocarditis, leading to severe insufficiency and rupture. Valve rupture is an emergency and should be managed as early as possible. The combination of aggressive antibiotic administration and surgical intervention is lifesaving in these cases. We emphasize the importance of timely diagnosis and prompt management from our case report.
